# Comparative analysis of platelet counts using Beckman Coulter DxH-900, Mindray BC-6800 Plus, CellaVision DM9600 and the flow cytometry reference method: addressing the challenge of giant platelets

**DOI:** 10.1515/almed-2025-0133

**Published:** 2025-11-24

**Authors:** Álvaro Piedra-Aguilera, Alba Leis-Sestayo, Alicia Martínez-Iribarren, Laura Jiménez-Añón, Jennifer Rodríguez-Domínguez, Ghali Ech Cherif-El Kettani, Xavier Tejedor-Ganduxé, Rebeca Jurado-Tapiador, Cristian Morales-Indiano

**Affiliations:** Clinical Analysis and Biochemistry Department, Germans Trias i Pujol University Hospital, Badalona, Spain; Hematology Laboratory, ICO-IJC-Hospital Germans Trias I Pujol, UAB, Badalona, Spain

**Keywords:** flow cytometry, giant platelets, impedance, optical method, platelet count, thrombocytopenia

## Abstract

**Objectives:**

To evaluate the analytical performance of platelet counts obtained by impedance- and optical-based methods on the DxH-900 and BC-6800 Plus analyzers, and by digital morphology using the CellaVision DM9600, in comparison with the international reference method (IRM), in samples with thrombocytopenia and both normal-sized and giant platelets.

**Methods:**

Platelet counts were analyzed in peripheral blood samples from patients with thrombocytopenia and normal platelet counts, including cases with normal-sized and giant platelets. Each sample was tested using two automated analyzers: the DxH-900 (impedance) and the BC-6800 Plus (impedance and optical). Results were compared with the IRM based on flow cytometry, performed on the DxFlex analyzer. Precision was assessed. The methods were compared by Passing-Bablok regression and bias calculation. Additionally, platelet morphology was reviewed digitally using CellaVision DM9600.

**Results:**

All methods demonstrated high precision and strong correlation with IRM in samples with normal-sized platelets. However, significant discrepancies were observed in the presence of giant platelets. Impedance methods showed higher imprecision (CV>10 %) and underestimated platelet counts with a negative bias exceeding −25 %. Conversely, the optical method showed better correlation (r=0.9888) and precision (CV=1.6 %), although it overestimated counts with a positive bias of 19.4 %. Digital morphology also demonstrated strong agreement with IRM.

**Conclusions:**

These findings highlight the analytical limitations of routine technologies in challenging samples and emphasize the importance of method selection for accurate platelet reporting. A correction factor for optical counts and the integration of digital morphology could enhance diagnostic reliability in cases involving giant platelets.

## Introduction

Accurate platelet quantification is essential in clinical laboratories for diagnosing and monitoring disorders related to platelet production, destruction or distribution, as well as for assessing bleeding risk, particularly in patients with hematologic and oncologic diseases. Although transfusion safety has improved considerably over recent decades, the use of blood products may involve important clinical and logistical considerations, including the risk of alloimmunization, transfusion reactions and transmission of infectious agents, as well as high costs and limited availability [1], 2]. Therefore, it is essential that transfusions are administered only when truly necessary. This underscores the need for highly accurate and precise platelet counts, as they directly impact clinical decision-making regarding transfusion support.

The impedance method remains one of the most widely used in clinical laboratories due to its simplicity and low cost. It is based on the detection of changes in electrical resistance as cells suspended in an electrolyte solution pass through a small aperture. Each cell generates a pulse whose amplitude is directly proportional to the cell´s volume, enabling the method to count and size individual cells with precision. However, this method has intrinsic limitations. It cannot distinguish particles of similar size to platelets, such as small or fragmented red cells. These interfering particles may be erroneously included in the platelet count, potentially leading to an overestimation of the true platelet number, which is especially critical in samples with severe thrombocytopenia. Conversely, large or giant platelets and platelet aggregation may be excluded from the count and may lead to falsely decreased counts [3]. This issue is particularly relevant in patients with immune thrombocytopenic purpura, myelodysplastic syndromes, or congenital macrothrombocytopenias, where large and giant platelets are frequently observed. To overcome the inherent limitations of impedance technology, several manufacturers have developed additional methods, such as optical and fluorescence-based methods, to improve the accuracy and reliability of platelet counting [4], [5], [6], [7], [8], [9], [10]. The incorporation of nucleic acid-binding fluorescent dyes enhances platelet identification and helps minimize interference from small red blood cells and cellular debris although it is not exempt from some limitations [3], 6].

In 2001, the International Council for Standardization in Hematology (ICSH) and the International Society of Laboratory Hematology (ISLH) recommended the International reference method (IRM) for platelet counting based on flow cytometry [11]. Although flow cytometry offers high specificity and reproducibility, its routine application is limited by technical complexity, manual procedures and high operational costs, making it unsuitable for use as a routine method to quantify platelets in general hematology laboratories [7].

The aim was to evaluate the analytical performance of impedance and optical platelet counting compared to the flow cytometry reference method, in samples with thrombocytopenia, normal-sized and giant platelets.

## Materials and methods

We evaluated the performance of different platelet counting methods: impedance on the Beckman Coulter DxH900 (Miami, FL, USA) (DxH-PLTi), and both impedance and optical methods on the Mindray BC-6800 Plus (Shenzhen, China) (BC-PLTi and BC-PLTo), comparing them with the flow cytometer Beckman Coulter DxFlex (Miami, FL, USA) (IRM) in samples with normal platelets counts, thrombocytopenia and samples with giant platelets. Furthermore, in samples with giant platelets, counts were obtained by digital morphology using the CellaVision DM9600. This system estimates the platelet number per high-power field and applies a multiplication factor of 10.4, previously defined by the laboratory based on correlation with the DxH900 in samples without platelet-related flags, covering the entire analytical range [12].

A total of 93 blood samples collected in K_3_-EDTA tubes between May 2024 and March 2025 were selected and divided into three groups: (a) normal-sized platelets >100×10^9^/L (n=30), (b) normal-sized platelets <100×10^9^/L (n=30), and c) giant platelets <100×10^9^/L, corresponding to patients with immune thrombocytopenic purpura, myelodysplastic syndromes or congenital macrothrombocytopenias (n=33). The inclusion criteria for selecting normal-sized platelets were a normal platelet histogram and the absence of platelet related-flags on the hematology analyzer (Supplementary Material, Figure 1A). Samples with giant platelets were selected based on an abnormal platelet histogram or the presence of analyzer flags (giant platelets and/or RBC–platelet histogram overlap). Giant platelets were confirmed by peripheral blood smear review by two expert observers. Samples were included when both observers agreed on their presence and the DM9600 count was ≥5 giant platelets per 100 leukocytes (Supplementary Material, Figure 1B).

Samples were processed by triplicate within 8 h after collection using the DxH900, BC-6800 Plus and DxFlex analyzers. The IRM was performed according to recommendations from the International Council for Standardization in Hematology (ICSH) and the International Society of Laboratory Hematology (ISLH) using two platelet monoclonal antibodies, CD41 and CD61 [11]. The study was approved by Ethics Committee of Hospital (PI-21-266).

To evaluate imprecision and calculate coefficient of variation (CV,%) each sample was analyzed by triplicate using the different methods. The percentage differences (bias) were calculated as: bias = (Cx – Cn) × 100/Cn (Cx: analyzer platelet count, Cn: flow platelet count). Comparisons between methods were assessed by Passing-Bablok regression analysis. Statistical analysis was performed using MedCalc version 19.6 software.

## Results

In samples with normal-sized platelets, all methods except BC-PLTi, showed good precision, with coefficients of variation <5.7 % (minimal specification established by the European Federation of Clinical Chemistry and Laboratory Medicine (EFLM)) in both normal and thrombocytopenic ranges (Table 1A, B). For platelet counts >100 × 10^9^/L, bias was 11.6 % for DxH-PLTi, 7.5 % for BC-PLTi, and 18.4 % for BC-PLTo, while for counts <100×10^9^/L, bias was 10.0 %, −1.9 %, and 9.1 %, respectively. All methods showed excellent agreement with flow cytometry, with correlation coefficients r>0.985 (Table 2A).

**Table 1:** Imprecision of platelet counting methods and mean platelet volume, stratified by platelet counts (A) >100 ×10^9^/L, (B) <100 ×10^9^/L and (C) samples with giant platelets and counts<100 ×10^9^/L.

(A)	Method	Mean ± SD 10^9^/L	% CV
Platelets >100×10^9^/L			
	IRM	304.6 ± 203.5	2.6
	DxH900-I	339.9 ± 231.7	1.5
	BC-6800 Plus-O	360.7 ± 249.8	1.6
	BC-6800 Plus-I	325.8 ± 222.5	2.3
			
MPV, fL	Beckman	8.6 ± 1.3	1.1
	Mindray	9.9 ± 1.6	1.1

(B)	Method	Mean ± SD 10^9^/L	% CV
Platelets <100×10^9^/L			
	IRM	41.5 ± 27.1	3.8
	DxH900-I	44.5 ± 27.7	4.2
	BC-6800 Plus-O	45.4 ± 29.4	3.2
	BC-6800 Plus-I	41.7 ± 27.1	7.8
			
MPV, fL	Beckman	9.1 ± 1.0	2.4
	Mindray	10.3 ± 1.2	4.2

(C)	Method	Mean ± SD 10^9^/L	% CV
Giant platelets <100×10^9^/L			
	IRM	48.3 ± 29.0	3.8
	DxH900-I	36.0 ± 25.9	16.3
	BC-6800 Plus-O	56.9 ± 33.3	1.6
	BC-6800 Plus-I	34.4 ± 24.5	11.5
			
MPV, fL	Beckman	11.1 ± 2.0	9.0
	Mindray	12.1 ± 1.6	5.7

IRM, international reference method; SD, standard deviation; CV, coefficient of variation; MPV, mean platelet volume.

**Table 2:** Comparison of platelet counts obtained by different methods in samples with A) normal sized platelets and B) giant platelets.

(A) Normal-sized platelets						
Method	n	Equation	95 % CI for intercept	95 % CI for slope	r	Bias, %
>100×10^9^/L						

DxH900-I vs. IRM	30	y = −0.4850 + 1.1154x	−13.2569 to 9.2523	1.0751 to 1.1893	0.9932	11.6
BC-6800 Plus-O vs. IRM	30	y =0.4322 + 1.1831x	−14.7788 to 18.9335	1.0926 to 1.2642	0.9869	18.4
BC-6800 Plus-I vs. IRM	30	y =10.5101 + 1.0243x	−11.5251 to 25.9807	0.9596 to 1.1180	0.9864	7.5

<100×10^9^/L						

DxH900-I vs. IRM	30	y =1.0757 + 1.0515x	−0.1909 to 2.6939	1.0104 to 1.1025	0.9936	10.0
BC-6800 Plus-O vs. IRM	30	y = −0.2003 + 1.0975x	−1.2209 to 1.5571	1.0459 to 1.1458	0.9949	9.1
BC-6800 Plus-I vs. IRM	30	y = −0.937 + 1.0169x	−1.6847 to 0.6107	0.966 to 1.0475	0.9927	−1.9

(B) Giant platelets						
Method	n	Equation	95 % CI for intercept	95 % CI for slope	r	Bias, %
<100×10^9^/L						

DxH900-I vs. IRM	33	y = −2.2633 + 0.8593x	−8.4896 to 0.9718	0.7610 to 0.9718	0.9042	−26.2
BC-6800 Plus-O vs. IRM	33	y =0.5106 + 1.1579x	−1.7112 to 2.6690	1.0883 to 1.2271	0.9888	19.4
BC-6800 Plus-I vs. IRM	33	y = −2.9061 + 0.8083x	−8.3791 to 1.4829	0.7050 to 0.9173	0.9324	−30.4
CellaVision DM9600 vs. IRM	32	y = −3.0957 + 1.1911x	−8.4370 to 1.6142	1.0479 to 1.3257	0.9311	13.9

IRM, international reference method; CI, confidence interval.

In presence of giant platelets, optical method maintained a strong correlation with the IRM (r=0.9888) and both the optical and flow cytometry demonstrated a good precision (1.6 and 3.8 %, respectively) (Table 1C). In contrast, impedance-based methods exhibited lower correlation with the IRM (r<0.940) and poor precision (16.3 % for DxH-PLTi and 11.5 % for BC-PLTi) (Table 1C and 2B), far above the minimal specification of 5.7 % established by the EFLM. The presence of giant platelets revealed an increased bias in impedance and optical technologies. The impedance method demonstrated a negative bias of −26.2 % and −30.4 % in the DxH900 and BC-6800 analyzers, respectively. Conversely, the optical method showed a positive bias of 19.4 %. Additionally, we compared the quantification of samples with giant platelets using digital morphology with the reference method. The observed bias was 13.9 % with a correlation coefficient of 0.9311 (Table 2B).

Mean platelet volume (MPV) and its imprecision were assessed in each group. Compared with normal-sized platelets, samples with giant platelets showed higher MPV values, exceeding a mean value of 11 fL with Beckman technology and 12 fL with Mindray technology. In samples with normal-sized platelets and counts >100×10^9^/L, the CV was <1.7 %, meeting the minimal specification. In contrast, the CV was >2 % in thrombocytopenic samples and >5 % in those with giant platelets (Table 1).

## Discussion

Within samples presenting normal-sized platelets, the impedance, optical and IRM methods demonstrated low imprecision, consistent with findings reported in previous studies [5]. In samples containing giant platelets, the impedance method exhibited greater imprecision, whereas the optical and IRM methods maintained CV values <5.7 %.

In thrombocytopenic samples with normal-sized platelets, bias was ≤10 % across all three technologies, consistent with previous reports [5], 6]. In contrast, the presence of giant platelets revealed marked discrepancies among methodologies. While earlier studies [4], 8] described lower bias in impedance and optical methods vs. IRM, their selection criteria differed: Guo et al. (2021) identified samples with MPV >15 fL and >30 giant platelets, whereas Prompetchara et al. (2024) selected those with platelet diameter >7 μm and >5 giant platelets per 200 WBCs, confirmed by digital morphology. These discrepancies highlight the lack of standardized criteria to select giant platelets and the limitations of MPV, which is analyzer-dependent, reflects only average platelet size, and may diverge from smear findings. Moreover, its imprecision did not meet the minimal EFLM specification. Both studies also evaluated interference from microcytic and fragmented red cells, which was not considered in the present work.

Considering the limitations of both impedance and optical methods, underestimation or overestimation of giant platelet counts should be reported by clinical laboratories. We suggest designing algorithms for routine implementation that report impedance platelet counts for patients with normal-sized platelets, but extend the optical platelet count for thrombocytopenic patients with suspected giant platelets. Despite the optical method demonstrated superior accuracy and strong correlation in the presence of giant platelets, the observed positive bias indicates that applying a correction factor may be necessary to ensure reliable platelet counts. Additionally, manual platelet counts performed via digital morphology demonstrated good correlation with the reference method and exhibited lower bias compared to other techniques, reinforcing its role as a valuable complementary tool.

Although flow cytometry based platelet analysis has been implemented in specialized centers, it remains uncommon in routine clinical laboratories due to persistent challenges in standardizing protocols and instrumentation, as well as the significant analytical complexity and inherent variability of platelet populations. As a result, alternative methods such as fluorescence-based approaches, digital morphology, or next-generation automated cytometers may provide more practical and broadly applicable solutions, although they still require thorough evaluation before routine implementation, particularly due to the lack of studies in samples with giant platelets [7], 9], 13], 14].

## Conclusions

In conclusion, for patients with giant platelets, the optical method demonstrated superior performance compared to impedance, although it exhibited a notable positive bias. To ensure accurate and reliable results, each laboratory must evaluate the analytical limitations of their hematology analyzers and understand the precision and accuracy of the methods in use. Familiarity with these technological pitfalls is essential for selecting the most appropriate approach to platelet quantification in complex cases. The implementation of algorithm-based decision tools, supported by digital morphology, may further enhance result accuracy in such challenging scenarios.

## Supplementary Material

Supplementary Material
