# Supplementary material for: Comparative analysis of platelet counts using Beckman Coulter DxH-900, Mindray BC-6800 Plus, CellaVision DM9600 and the flow cytometry reference method: addressing the challenge of giant platelets
Source: Adv Lab Med. 2025 Nov 24;7(1):59–63. doi: 10.1515/almed-2025-0133 (PMC12994698; doi:10.1515/almed-2025-0133)
Supplement: Supplementary file 1 — Supplementary Material [file j_almed-2025-0133_suppl_001.docx]

**Fig. 1. Supplementary.** Histograms (upper panel) and digital morphology images (lower panel) of peripheral blood smears showing normal-sized (A) and giant platelets (B).

A)


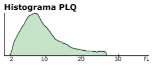

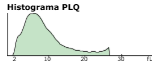

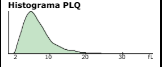

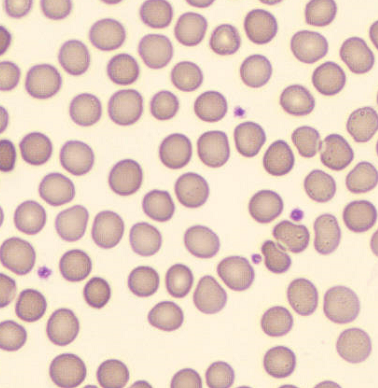

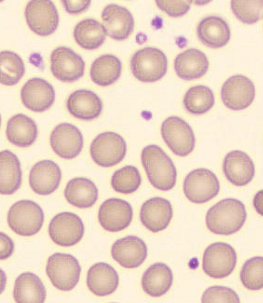

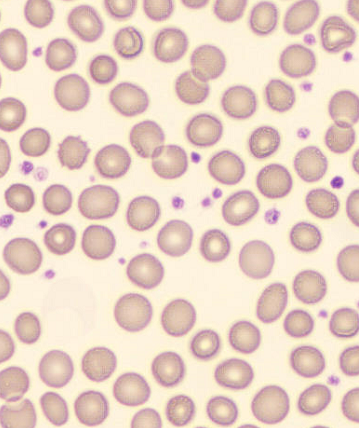


**Platelet count**

**Platelet count**

**Platelet count**

**2**

**10**

**20**

**30**

**fL**

**2**

**10**

**20**

**30**

**fL**

**2**

**10**

**20**

**30**

**fL**

B)

**2**

**10**

**20**

**30**

**fL**

**2**

**10**

**20**

**30**

**fL**


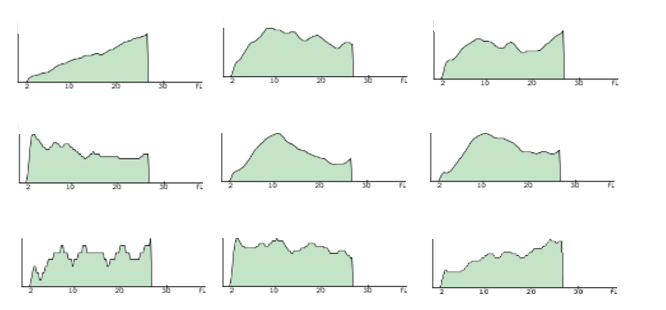

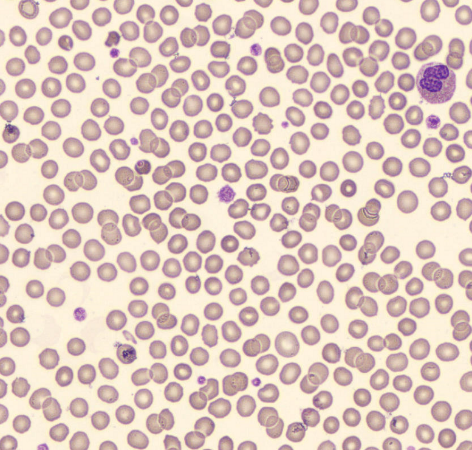

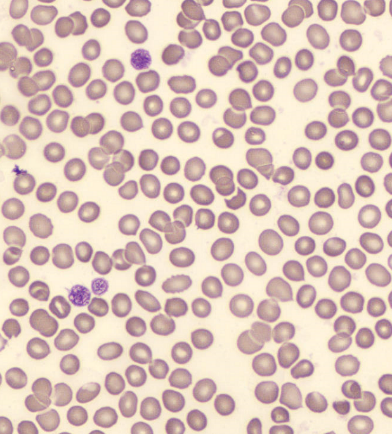

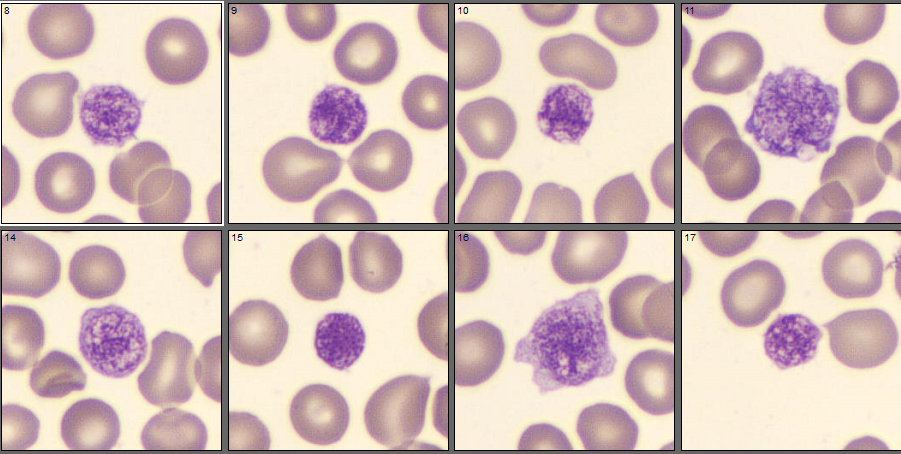


**2**

**10**

**20**

**30**

**fL**

**Platelet count**

**Platelet count**

**Platelet count**

Abbreviations: fL, femtoliter.
